# Delayed administration of ixazomib modifies the immune response and prevents chronic graft-versus-host disease

**DOI:** 10.1038/s41409-021-01452-1

**Published:** 2021-09-23

**Authors:** Teresa Lopes Ramos, Estefanía García-Guerrero, Teresa Caballero-Velázquez, Alfonso Rodríguez-Gil, Rocío Caracuel-García, Melanie Nufer, María José Robles-Frías, María Victoria Barbado, José A. Pérez-Simón

**Affiliations:** 1 Instituto de Biomedicina de Sevilla (IBIS/CSIC), CIBERONC, Universidad de Sevilla, Sevilla, Spain; 2 Division of Blood and Marrow Transplantation, Stanford University School of Medicine, Stanford, CA USA; 3 Department of Hematology, University Hospital Virgen del Rocio, Universidad de Sevilla, Sevilla, Spain

**Keywords:** Bone marrow transplantation, Translational research

## Abstract

In this study, we aimed to modify the immune response in the long term after allogeneic bone marrow transplantation (allo-BMT) by using the proteasome inhibitor ixazomib (IXZ) at the late stages of the post-transplant period. This approach facilitated the immune reconstitution after transplantation. IXZ significantly prolonged survival and decreased the risk of chronic graft-versus-host disease (cGvHD) in two different murine models without hampering the graft-versus-leukemia (GvL) effect, as confirmed by bioluminescence assays. Remarkably, the use of IXZ was related to an increase of regulatory T cells both in peripheral blood and in the GvHD target organs and a decrease of effector donor T cells. Regarding B cells, IXZ treated mice had faster recovery of B cells in PB and of pre-pro-B cells in the bone marrow. Mice receiving ixazomib had a lower number of neutrophils in the GvHD target organs as compared to the vehicle group. In summary, delayed administration of IXZ ameliorated cGvHD while preserving GvL and promoted a pro-tolerogenic immune response after allo-BMT.

## Introduction

Although survival rates have improved over the years, a large proportion of patients undergoing allogeneic hematopoietic stem cell transplantation (allo-HSCT) still develop graft-versus-host disease (GvHD) [1–4], which is the main cause of death and markedly affects the quality of life after allo-HSCT [5, 6]. The most effective strategies to prevent GvHD are based on the use of T cell depletion [7, 8]. The effectiveness of allo-HSCT is based on the allorecognition of donor T cells, which induces a cytotoxic effect on tumor cells from the host (graft-versus-leukemia effect, GvL). Thus, the same mechanism of allorecognition is responsible for both GvHD and GvL, therefore any procedure aimed to prevent GvHD by increasing immunosuppressive treatment might affect GvL [9, 10].

Overall, risk profiles are similar for both acute and chronic GvHD [11]. Prior aGvHD is the main risk factor to develop cGvHD, but other risk factors also play an important role, such as the impact of donor sex or age and the use of mobilized peripheral blood cells are risk factors of chronic but not for aGvHD [3, 4]

The pathogenesis of acute GvHD has been well characterized. In the case of chronic GvHD, the disease develops as a result of complex molecular/biology networks, from tissue damage to unusual antigen presentation and aberrant myeloid and lymphoid (T and B cell) interactions [12].

Recently, a 3-step model has been proposed to explain the pathophysiology of cGvHD. Phase I is the initial phase and is an effect of early post-transplant inflammation and tissue injury. The release of inflammatory cytokines activates antigen-presenting cells (APC) that consequently stimulate the activation of donor allo-reactive T cells with an enhanced Th1/Tc1 and Th17/Tc17 T cell effector lineages and macrophages sequestration in tissues. Phase II is characterized by the presence of chronic inflammation and dysregulation of the immune system that cannot be counterbalanced by the regulatory immune responses. The dysregulated immunity and aberrant tissue repair can lead to the propagation of fibrosis. Phase 3, aberrant repair mechanisms promote the release of profibrotic mediators by monocytes and macrophages, leading to fibroblast activation and collagen deposition and fibrosis [12–16].

The differences in the pathophysiology between acute and chronic GvHD might allow to design of specific approaches for cGvHD prophylaxis without increasing immune suppression or abrogating the alloreactive immune response early after transplantation. For this purpose, we aimed to target the cell subsets involved in cGvHD development. Remarkably, NF-κB plays a key role in the activation of T or B cells, macrophages, and dendritic cells [17]. Regarding lymphocytes, NF-κB is involved in pathways downstream T and B cell receptors [17–19]. APC cells also depend on NF-κB to become fully activated through Toll-like receptors (TLR) [17]. Proteasome inhibitors block the activation of NF-κB. Bortezomib (Bz) exerts a potent pro-apoptotic effect on activated T cells, preserving the viability of resting T-lymphocytes and regulatory T cells (Treg) [20–22], and prevent GvHD both in preclinical and in clinical models [23–25].

Next generations of proteasomes inhibitors have been developed with different toxicity profiles compared with bortezomib. Ixazomib (IXZ) is an orally bioavailable inhibitor of the 20S proteasome. This drug has similar selectivity and potency to Bz in biochemical and cell-based assays, but with the advantage that it has a shorter 20S proteasome dissociation half-life. In addition to NF-κB, proteasome inhibitors affect other pathways involved in immune cells activation and viability [26–28].

In this study, we aimed to challenge the classic approach of preventing cGvHD by focusing on the abrogation of the immune response in the early post-transplant period. Since most pathophysiological mechanisms leading to cGvHD are triggered early but are perpetuated late after transplant, we hypothesized that new approaches could be designed to modify the immune response at later stages of the transplant period to favor a tolerogenic immune response. For this purpose, we evaluated a delayed administration of IXZ confirming its efficacy in ameliorating the incidence of delay/progressive chronic GvHD without hampering GvL.

## Methods

### Mice

BALB/c (H-2^d^) and C57BL/6 (H-2^b^) and B10.D2 (H-2^d^) mice were purchased from Charles River Laboratories (Morrisville, NC). The green fluorescent protein (GFP) C57Bl/6-Tg(ACTB-EGFP)1Osb/J) (H-2^b^) [29] were housed in the animal facility. All experiments were performed with sex-matched animals and mice were between 7–10 weeks of age. Mice were held under specific pathogen-free conditions. Animal protocols were approved by the Institutional Animal Care and Use Committee at the Institute of Biomedicine in Seville.

### GvHD model

For progressive chronic GvHD (pcGvHD), recipient mice (BALB/c) were irradiated with 8.6 Gy split into 2 doses. To induce GvHD, irradiated recipients received 5 × 10^6^ bone marrow (BM) cells and 2 × 10^6^ donor splenocytes (SC) cells, previously cultured at 37 °C for 2 h from C57Bl/6-Tg donor on day 0. This model was based on previous reports [16, 30, 31] which described cGvHD features in the long term by infusing a low number of splenocytes. In our model, the recipient mice showed signs of acute GvHD starting around day +20 post-transplantation. Around day +49 surviving mice started to gain weight and develop new skin lesions and alopecia. Histological examination confirmed the presence of chronic signals in the different target organs (Supplementary Fig. 1).

For the sclerodermatous cGvHD model (scGvHD), BALB/c mice received lethal total body irradiation (10 Gy) split into 2 doses of 5 Gy, 24 h apart. BM cells (10 × 10^6^ cells) with SC cells (15 × 10^6^ cells) from B10.D2 donor mice (H2^d^) were intravenously infused into lethally irradiated, major MHC-identical, minor histocompatibility antigen-mismatched recipient BALB/c (H2^d^) mice.

Recipients were monitored daily, body weight and GvHD signs were assessed twice applying standardized scoring. The scoring system includes the following parameters: weight loss, posture, activity, fur texture, and skin integrity (maximum index of 10). The criteria for euthanasia were based on clinical scores and tumor-related symptoms [23, 32]. Prior studies have shown that in acute/chronic GvHD model cohorts of 5–8 animals per group. To detect the significant impact of the treatment we elected to perform three independent experiments with 8 to 12 mice per group. Mice reaching a GvHD score of 8/10 were sacrificed in agreement with the request of the ethical committee. The scores and weight of these animals were kept in the data set and the euthanized animals were counted as dead in the survival curves. The mice that died before starting the treatment were excluded from the experiment.

Ixazomib (IXZ) was dissolved in 5% of 2-hydroxypropyl-β–cyclodextrin (Sigma-Aldrich). IZX at 1 mg/Kg starting on day +21 for pcGvHD and at 3 mg/Kg from day +30 in the scGvHD model was administered subcutaneously two times per week until the end of the experiment. Cyclosporine A (CsA)-treated mice received 5 mg/kg intraperitoneally (IP) from day −1 until day 120 days post-transplant, five times a week. The control groups received vehicle solutions according to the same schedule as the treatment group. The technician without interest in the study outcome randomizes the mice for the experiment. To avoid bias, in the same cage there were animals that received the drug and others the vehicle. No blinding was done during the treatment and GvHD scorings.

### Graft-versus-leukemia assays and bioluminescence imaging

In vivo bioluminescence imaging was performed as previously described [33] with an IVIS Lumina III in vivo imaging system (PerkinElmer, Massachusetts, USA). Images were analyzed with Living Image Software 3.2 (Caliper Life Sciences). A20 (B cell lymphoma) cell line was acquired from American Tissue Culture Collection (ATCC; TIB-208) and transfected with luciferase and GFP. The cells were injected into recipient BALB/c mice intravenously at a dose of 0.5 × 10^6^ cells. A20 cells were injected at day 0 after BMT as described. Tumor growth was monitored once a week using an IVIS imaging system.

### Histopathological examination and tissue digestion

Detailed information, including tissue digestion and histology, is provided in supplementary methods.

### Flow cytometry assays

Flow cytometry analysis methodology is provided in supplementary methods.

### Statistics

For murine GvHD survival experiments, the differences in animal survival (Kaplan-Meier survival curves) were analyzed by the Mantel-Cox test.

For statistical analysis of 2 groups, an unpaired 2-tailed Student’s *t*-test was applied. All data were tested for normality applying the Kolmogorov-Smirnov test. If the data did not meet the criteria of normality, the Mann-Whitney *U* test was applied. When more than 2 groups were analyzed, we used the Kruskal-Wallis test if nonparametric testing was suggested, and we performed a 1-way ANOVA in case of normally distributed data.

All data are shown as mean ± standard error of the mean (SEM) and represent a combined data from at least three independent experiments. Figures were prepared using GraphPad Prism 5.

*P* values <0.05 (*), <0.001 (**), and <0.001 (***) were considered significant.

## Results

### Ixazomib decreases the risk and severity of progressive chronic GvHD and improves survival in a murine model

**Fig. 1:**
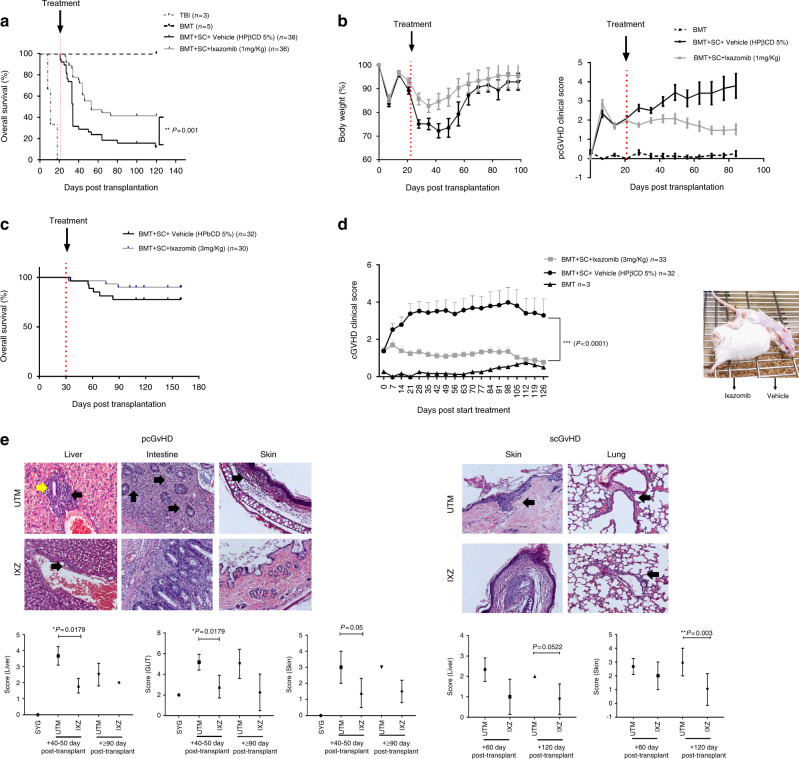
Ixazomib administration increases the survival and protects mice from progressive chronic and sclerodermatous chronic GvHD. Effect of IXZ on pcGvHD in a murine model. **A** Kaplan-Meier survival curves of mice receiving IXZ at 1 mg/Kg, two times a week subcutaneously from day +21 post-transplantation or vehicle. **B** clinical score and weight of mice receiving IXZ versus untreated transplanted mice (UTM). In all experiments, UTM received the vehicle of the drug. **C** For scGvHD, recipient BALB/c mice were randomized to receive vehicle (UTM) (HPβCD 5%) or IXZ (3 mg/Kg) subcutaneously from day +30 after transplant. Kaplan-Meier survival curves of mice receiving IXZ or vehicle (UTM) are shown. **D** represents the clinical score of mice receiving IXZ versus UTM. Photographs were taken on day +80 after HSCT from scGvHD mice treated with vehicle or IXZ. **E** Scores were evaluated in the intestine, colon, skin, and liver using a semi-quantitative score by pathologists in a blind code fashion. Histopathology analysis of liver, large and small bowel, skin, and liver samples from the different experimental groups are shown. In the pcGvHD it was observed the presence of lymphocytic infiltration in periportal areas (black arrows) with the loss of bile duct (yellow arrows), loss of crypts in the large bowel (black arrows), vacuolar degeneration in the skin (black arrows). scGvHD: Presence of fissures in the skin and the presence of perivascular inflammatory infiltrate in the lung. Data are shown as mean ± standard error of the mean (SEM). Data are collected from 4–5 independent experiments with 5–6 mice per group. Student’s *t*-test *P < .05, **P < .01, and ***P < .001 were considered significant. BMT: bone marrow transplantation; SC: splenocytes; TBI: total body irradiation. UTM: untreated transplanted mice (vehicle administration); IXZ: ixazomib group; HD: healthy donor; SYG: syngeneic group. Original magnification: ×200.

Using a GvHD model of C57BL/6 donor into MHC-mismatched BALB/c recipient, we evaluated the effect of Ixazomib (IXZ) on pcGvHD (Fig. 1). Treatment with IXZ significantly prolonged survival as compared to those mice that received only vehicle (designed as untreated transplanted mice: UTM) (*P* = 0.001) (Fig. 1A). IXZ treatment also attenuated the clinical signs of GvHD and weight loss (Fig. 1B). Next, we evaluated the combination of IXZ plus cyclosporine A (CsA) at 5 mg/Kg, the latter being started on d-1. All treatment groups had improved survival when compared with the UTM, the combination being significantly better as compared to either drug alone (Supplementary Fig. 2). Prolonged administration (14 weeks) of IXZ did not delay platelet or white blood cell recovery after transplantation (Supplementary Fig. 3), thus showing that IXZ administration efficiently protected mice from pcGvHD without significant myelotoxicity.

Next, we used a murine model of sclerodermatous cGvHD (scGvHD) [34]. Recipient mice were treated either with vehicle (UTM) or IXZ from day +30 after transplantation, the time when the signs of scGvHD start. Treatment with IXZ significantly decreased the formation of skin lesions, alopecia, and mobility score, with a significant reduction of the overall cGvHD score when compared to UTM (Fig. 1C, D).

Histologic features were analyzed in both models in GvHD target organs. Regarding pcGvHD, large bowel crypt structures were severely disturbed with an increased number of apoptotic bodies, cryptal abscesses, and the presence of inflammatory cells. In the liver, ductopenia was observed together with diffuse microvesicular steatosis with lymphocytic infiltration in periportal areas. In the skin, fibrosis and atrophy of adnexal structures were observed. Treatment with IXZ significantly decreased the overall GvHD histopathological score (Fig. 1E) on day 40–50 post-transplantation, with the most pronounced differences observed in the gut (*P* = 0.0179) and liver (*P* = 0.0179). As far as the histopathological score in the scGvHD model is concerned, phenotypic hallmarks of cGvHD were also observed in the skin, with epidermal atrophy, dermal fat loss, hair follicle destruction, and mononuclear cell infiltration. The other target organs analyzed also showed pathological involvement. IXZ decreased histopathological scores in both times points analyzed (Fig. 1E). These results confirmed a protective effect of IXZ on cGvHD development.

### The use of Ixazomib was associated with a faster recovery of a normal pattern of T cell subpopulations post-transplant and a significant increase of regulatory T cells

Once we confirmed the effect of IXZ on the pcGvHD in vivo, we analyzed the different immune cell subpopulations reconstitution once donor complete chimerism was confirmed, as assessed by the presence of donor cells (GFP+) (Supplementary Fig. 4)

**Fig. 2:**
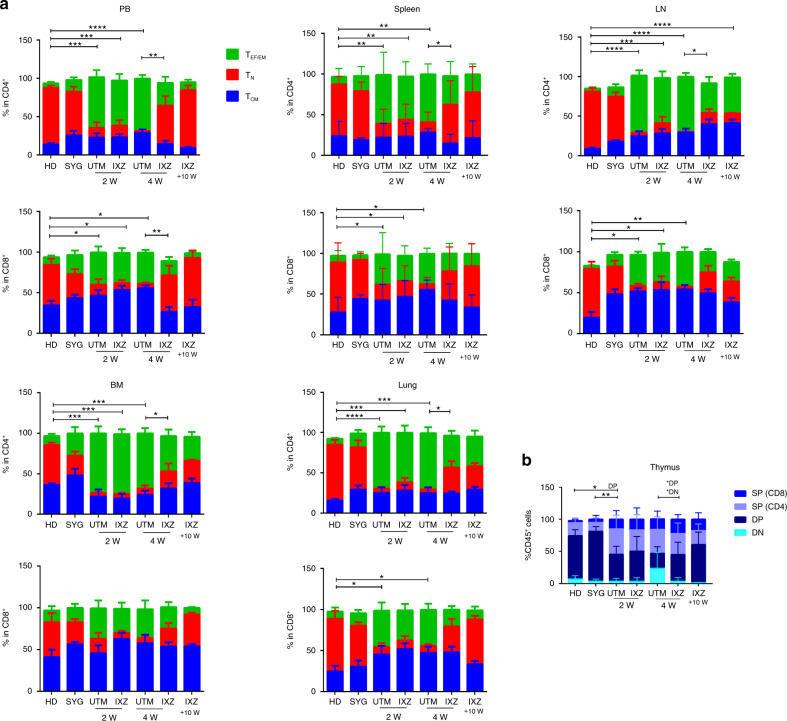
Ixazomib treatment significantly decreases the T effector cells in pcGvHD model. Flow cytometry assays of the different organs at different time points after treatment were performed. **A** Percentage of effector (T_EF/EM_:CD44^++^CD62L^−^) T cells were analyzed. **B** Percentage of CD8^+^ (SP), CD4^+^ (SP), CD4^+^ CD8^+^ (DP) and CD4^−^CD8^−^ (DP) in the thymus. Data are shown as mean ± SEM. Data are collected from 4–5 independent experiments with 5–6 mice per group. *P < .05, **P < .01, and ***P < .001. UTM: untreated transplanted mice (vehicle administration); IXZ: ixazomib group; HD: healthy donor; SYG: syngeneic group; 2/4 and +10 W: weeks after starting the treatment. *P* values were calculated using 1-way ANOVA and 2-sided Student’s unpaired *t*-test.

Upon analyzing the expression of CD44 and CD62L in CD4^+^ and CD8^+^ T cells, we evaluated naïve, effector, and memory T cell subpopulations along with transplantation. We observed an increase of effector T cells (T_EF/EM_) after allo-transplant cells when compared to healthy donors (HD) and mice transplanted from a syngeneic donor (SYG). After 4 weeks of IXZ treatment, a significant decrease of CD4^+^ and CD8^+^ effector T cells in the PB, spleen, and LN was observed when compared with the group that received vehicle (UTM). Similar behavior was observed in the other GvHD target organs analyzed (Fig. 2A). Interestingly, 10 weeks after starting treatment, mice receiving IXZ displayed a similar pattern of effector cells as compared to the HD and SYG groups, with the exception of the LN. There was still observed an increase of effector CD4^+^ T cells. In the spleen, a decrease of effector CD4^+^ T cells was followed by a decrease of IFN-γ (Supplementary Fig. 5).

In addition, a significant reduction in the percentage of CD4^+^ CD8^+^ thymocytes (DP) was observed in UTM mice, a distinctive hallmark of thymus GvHD [35]. IXZ group showed a significant increase of DP as compared with UTM (Fig. 2B).

**Fig. 3:**
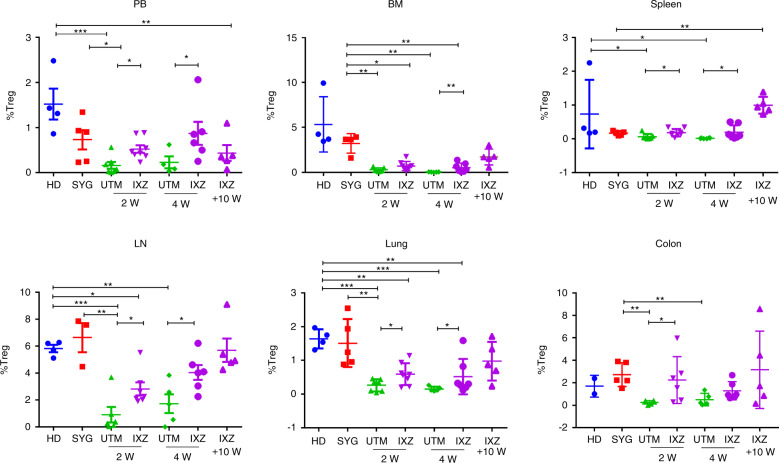
Ixazomib treatment significantly increases the regulatory T cells. Percentage of Treg in CD45^+^ population (CD45^+^ CD3^+^ CD4^+^ CD25^+^ Foxp3^+^) in the different organs analyzed. Data are shown as mean ± SEM. Data are collected from 4–5 independent experiments with 5–6 mice per group. *P < .05, **P < .01, and ***P < .001. UTM: untreated transplanted mice (vehicle administration); IXZ: ixazomib group; HD: healthy donor; SYG: syngeneic group; 2/4 and +10 W: weeks after starting the treatment. *P* values were calculated using 1-way ANOVA and 2-sided Student’s unpaired *t*-test.

We and others have previously described that proteasome inhibitors can purge alloreactive T cells but preserve regulatory T cells (Treg) [21]. Interestingly, treatment with IXZ significantly increased the percentage of CD45^+^ CD3^+^ CD4^+^ CD25^+^ FOXP3^+^ cells in PB and in the different organs analyzed (Fig. 3). The recovery of the regulatory T cells was confirmed in the IXZ group along the 10 weeks of follow-up.

### Ixazomib enhances B cell recovery after transplantation and increases the Breg

**Fig. 4:**
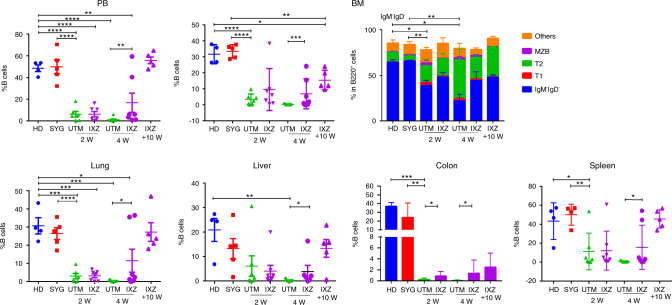
Ixazomib treatment increases the recovery of B cells. Flow cytometry analysis of B cells (CD45^+^ CD19^+^) in the PB and in the different organs. The distribution of the different B cell maturation in the BM panel represents the results from pcGVHD. Data are shown as mean ± SEM. Data are collected from 4–5 independent experiments with 5–6 mice per group. *P < .05, **P < .01, and ***P < .001. 2-sided Student’s unpaired *t*-test UTM: untreated transplanted mice (vehicle administration); IXZ: ixazomib group; HD: healthy donor; SYG: syngeneic group; 2/4 and +10 W: weeks after starting the treatment. MZB: Marginal Zone B Cells B220^+^ CD23^− /low^CD21^+^; T1: transitional T1 (B220^+^ CD21^+^ IgM^+^); T2: transitional T2 ^(^B220^+^ CD21^−^ IgM^+^).

B cell reconstitution takes weeks to months after allo-transplantation, with limited BCR diversity posttransplant [36]. In the pcGvHD model, we observed a decreased percentage of B cells (CD45^+^ CD19^+^) after transplant in the untreated mice (Fig. 4). Remarkably, mice receiving IXZ showed a significantly faster recovery in the percentage of CD19^+^ cells after 4 weeks in the different target organs analyzed, which was maintained at the long term (+10 weeks), Fig. 4A. Some authors have described a decrease of B lineage-specific hematopoietic progenitor cells (CD34^+^ CD19^+^) in cGvHD [37]. In our studies, the percentage of pre-pro-B cells (phenotypically defined as B220^+^ IgM^−^IgD^−^) in the BM was significantly decreased after allo-BMT. IXZ treated mice showed a significantly higher percentage of progenitor B cells as compared with UTM (Fig. 4).

### Ixazomib treatment modifies the recovery of myeloid cells post-transplant

**Fig. 5:**
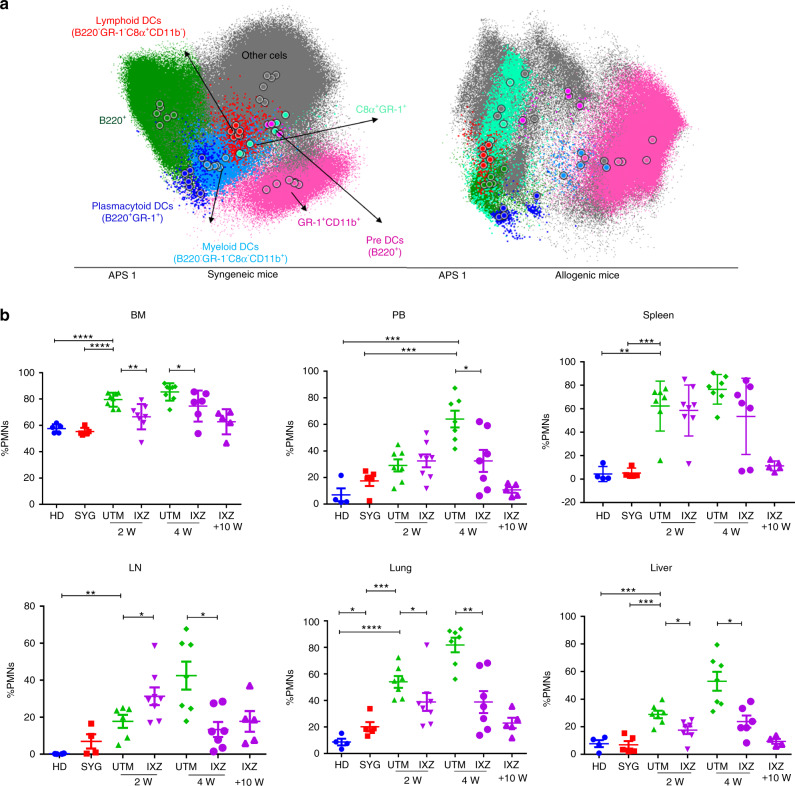
Myeloid population after transplantation in pcGvHD model. Flow cytometry assays of the different organs at different time points after treatment were performed. **a** Automatic population separation (APS) representative figure of HD and UTM. **b** PMNs (GR-1^+^CD11b^+^) in the different GvHD target organs. Data are collected from 4–5 independent experiments with 5–6 mice per group. Data are shown as mean ± SEM. *P < .05, **P < .01, and ***P < .001. *P* values were calculated using 1-way ANOVA (**b**). UTM: untreated transplanted mice (vehicle administration); IXZ: ixazomib group; HD: healthy donor; SYG: syngeneic group; 2/4 and +10 W: weeks after starting the treatment.**;** PMNs: polymorphonuclear cells.

Myeloid cell populations including dendritic cells (DCs), neutrophils, macrophages, and certain monocytes subsets play a key role in the pathophysiology of GvHD. Using GR and CD11b markers we analyzed the percentage of polymorphonuclear cells (PMNs). After allo-transplantation, a significant increase of PMNs on the lymphoid and non-lymphoid organs was observed (Fig. 5). Interestingly, ixazomib treatment decreased the percentage of PMNs cells infiltrating the liver (*P* = 0.0140) and lung (*P* = 0.082) after 4 weeks of treatment or even sooner in some organs. The percentage of PMNs was similar in the IXZ group after 10 weeks of treatment as compared to the HD and SGY groups.

### Ixazomib decreases GvHD but maintains graft-versus-leukemia activity

**Fig. 6:**
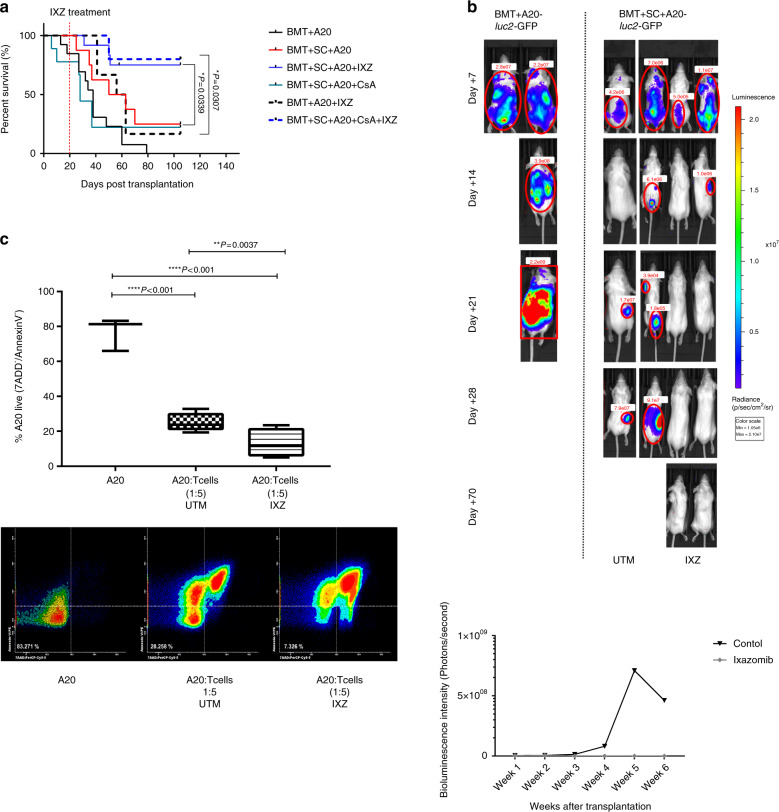
Ixazomib administration protects mice from pcGvHD while maintaining a potent GvL effect. Irradiated BALB/c mice were transplanted with BM and A20 luciferase-transfected lymphoma cells with or without spleen cells at day 0. (**A**) Survival curves of mice receiving IXZ at 1 mg/Kg, two times per week sc. from day +21 post-transplantation or vehicle (UTM), and IXZ plus cyclosporine A (CsA) (at 5 mg/Kg, intraperitoneally, 5 days a week from day −5) or CsA alone. **B** Bioluminescent images were acquired to monitor tumor burden. **C** Viability (annexin V^−^/ 7AAD^−^) of A20 cells that was co-cultured for 18 h with splenocytes (ration 1:5) obtained from UTM or IXZ treated mice transplanted with BM, spleen cells, and A20 cells. Data were collected from 2 independent experiments with 3–4 mice per group (*n* = 7–8). Data are shown as mean ± SEM. *P < .05, **P < .01, and ***P < .001. Survival data were plotted by the Kaplan-Meier method and analyzed by the log-rank test. *P* values were calculated using a one-way ANOVA test (**C**). UTM: untreated transplanted mice; IXZ: ixazomib group.

Once we confirmed the effect of IXZ, we aimed to determine its effect on graft-versus leukemia (GvL). For this purpose, mice were transplanted with A20-*luc2* lymphoma cells together with BM, and with or without splenocytes. The group transplanted with A20 plus BM showed the worst survival, with all mice dying before day +80 (Fig. 6A). The survival curve slightly increased among mice also receiving splenocytes (Fig. 6A, red line). In this subgroup, lymphoma cells were detected for up to 4 weeks (Fig. 6B). Treatment with IXZ significantly increased overall survival (BMT + SC + A20 vs. BMT + SC + A20 + IXZ *p = 0.0339). Remarkably, bioluminescence assays confirmed the elimination of tumor cells after 3 weeks among IXZ treated mice (Figs. 6A and 6B). Next, we performed the same experiments adding cyclosporine A. Treatment with cyclosporine alone did not improve survival after transplant; by contrast, a significant improvement was observed upon using CsA and IXZ (BMT + SC + A20 + CsA vs. BMT + SC + A20 + CsA + IXZ *p = 0.0307). The concentration of IXZ used in our study was not able (Fig. 6A, discontinued black line) to rescue the mice injected with BM + A20 in the absence of splenocytes.

To further confirm the GvL effect of the T cells, we co-cultured the spleen cells from the mice that were transplanted with BM + SC + A20 (with or without IXZ) with A20-GFP^+^ cells. We observed a significant decrease of A20 cells viability after exposure to T cells, which was more pronounced when A20 were co-cultured with lymphocytes from IXZ-treated mice (Fig. 6C).

Our results demonstrate that IXZ can successfully ameliorate GvHD while maintaining a potent GvL effect.

## Discussion

Current approaches for GvHD prophylaxis are focused on specific maneuvers aimed to eliminate donor T cells early after transplant, with the hope that immune tolerance will spontaneously arise in the absence of alloreactive T cells. Nevertheless, as far as the pathophysiology of cGvHD is concerned, many different players must be considered in addition to T cells. Thus, active approaches might be designed to induce immune tolerance in the long term, with the potential advantage of maintaining enough T cells from the donor capable to induce GvL. In our study, we hypothesized that IXZ treatment in the late posttransplant period ameliorates cGvHD, favors a protolerogenic immune reconstitution, and maintains GvL. While we and others have already reported the potential use of proteasome inhibitors in the GvHD setting, previous studies have focused on early post-transplant interventions. By contrast, in this study, we propose to evaluate whether it is possible to modulate the function of the different cell subsets involved in cGvHD pathophysiology, promoting a pro-tolerogenic immune response in the long term.

To test this hypothesis, we used a progressive chronic GvHD model applying a major histocompatibility antigen mismatched. The mice were transplanted with progenitor cells and low numbers of splenocytes. In this model, the mice started to develop the characteristic of acute clinical signs around day 21 posttransplant, and those surviving this acute phase subsequently stabilized their weight and develop chronic signals. This is in accordance with previous reports showing that low-dose donor spleen cells allow the mice to survive aGvHD, and promote the expansion of autoreactive T cells and the development of cGVHD [30]. Thus, these findings resemble the typical outcome of a progressive chronic GvHD in the clinical setting, which was confirmed in the histopathological examination. For the sclerodermatous cGvHD model, we applied a minor histocompatibility antigen mismatched model.

The progressive chronic GvHD mouse model recapitulates the immunologic features described after allo-HSCT [38]. More specifically, regarding T cells, in the pcGvHD model, we confirmed an abnormal CD4^+^ /CD8^+^ proportion and a significant increase in the effector T cell numbers, as previously described [39]. This pattern was not only observed in the PB, but also in GvHD target organs.

The thymus is the primary organ for tolerance induction and the development of T-lymphocytes. Previous studies have described that thymic damage after allo-HSCT results in a defective negative selection [40]. In our study, a significant reduction in the percentage of CD4^+^ CD8^+^ thymocytes (DP), a distinctive hallmark of thymus damage was observed in UTM mice [35]. IXZ group showed a significant increase in this population. GvHD is characterized by an imbalance between effector and regulatory immune function. Interestingly, ixazomib treatment in the context of chronic GvHD evolution was able to decrease the effector T cells and increase the percentage of regulatory cells. Our data confirm our previous studies describing the capacity of the proteasome inhibitor to preserve regulatory T cells while abrogating activated conventional T cells viability [21], but with this study, we showed this capacity also in the GvHD target organs.

In the post-HSCT, the alloantigen-rich microenvironment leads to aberrant activation of T and B cells, a characteristic signature of cGvHD [41].

As far as the B cell recovery is concerned, previous studies have shown that, within germinal centers, donor B cells produce autoantibodies which are responsible for the development of sclerotic manifestations of cGvHD [13, 42]. Different models have described the loss of the B cell pool in the BM and periphery during GvHD. Kolupaev et al. (2018) [43] found decreased numbers of common lymphoid progenitor (CLPs) pro-pre-B cells and immature B cells in the BM within a bronchiolitis obliterans syndrome (BOS) mouse model of cGvHD. The mechanism responsible for this abnormal B cell development was explained because of the donor CD4+ T cells infiltrating the BM. They also observed that Treg cells can revert the effects of donor T cells on B cell development in the BM. In our pcGvHD model, treatment with IXZ fostered B cell regeneration by increasing the percentage of pre-pro-B cells in BM. Again, these results agree with previous studies [43]. Remarkably, mice receiving IXZ reached a pattern like the control group significantly sooner than UTM both for B cells and for pre-pro-B cells.

Concerning myeloid populations, there is evidence that neutrophils play a key role in the pathophysiology of GvHD [44, 45]. Recent studies showed their multifaceted activities, where they can also function as key effectors in the innate and adaptative immune response with the capacity of prime T cells activation both in vitro and in vivo [46, 47]. In the acute GvHD animal model, the treatment with antibody mediates (anti-Ly6G) or genetically determined depletion of neutrophils was found to reduce aGvHD aggressiveness and mortality [48]. In chronic GvHD, neutrophils are hypothesized to be involved in ocular cGvHD pathogenesis in patients, mainly via their release of neutrophil extracellular traps (NETs) [49]. IXZ was able to decrease the number of neutrophils in the GvHD target organs, future studies will be important to address the effect of ixazomib on the eventual possibility of modulating their function in allo-HSCT.

In summary, we were able to ameliorate chronic GvHD while preserving GvL response by delaying IXZ administration. Ixazomib treatment was able to modify the immune system with a faster recovery of the different cell subpopulations analyzed favoring a more pro-tolerogenic immune response. An ongoing clinical trial aims to determine the effects of ixazomib in patients undergoing allo-HSCT.

## Supplementary information

Supplemental Material
