# Supplementary material for: Delayed administration of ixazomib modifies the immune response and prevents chronic graft-versus-host disease
Source: Bone Marrow Transplant. 2021 Sep 23;56(12):3049–58. doi: 10.1038/s41409-021-01452-1 (PMC8636253; doi:10.1038/s41409-021-01452-1)
Supplement: Supplementary file 1 — Supplemental Material [file 41409_2021_1452_MOESM1_ESM.docx]

**Supplemental material**

**Histopathological examination and tissue digestion**

Single-cell suspensions of spleen were prepared by passing the organs through a cell strainer. BM were harvested from the bones. Lung, liver, LN, thymus, and payer patches were digested for 30-40 min in medium supplemented with collagenase IV and DNase (Gibco Invitrogen Corporation and, Roche Applied Science, Indianapolis, IN, respectively). After digestion, the different organs were passed through a cell strainer (40 µm). In the liver, the leukocytes were isolated using a percoll (Sigma-Aldrich) gradient centrifugation.

The colon was removed, cut longitudinally, and washed three times with PBS. The pieces were incubated under stirring in Ca/Mg-free PBS containing 5 mM EDTA (Chem-Supply) and 1mM DTT for 30 min at 37°C. After removing the intraepithelial leukocytes, the tissue was incubated for 30 min at 37°C in RPMI including 5 μg/ml DNase + 5 μg/ml Collagenase 4 (Sigma-Aldrich) and then passed through a 100-μm cell strainer to obtain the lamina propria leukocyte (LPL) fraction. For skin was used a solution of dispase (Ginco), liberase (Roche) and DNase. After digestion, the cells were filtered through a 100-µm strainer.

Skin, small and large intestine, lung and liver from recipient mice were fixed in 10% formalin, embedded in paraffin, and cut into 5-mm-thick sections for hematoxylin and eosin (H&E, both from Merck KGaA, Darmstadt, Germany) staining. In blinded fashion, slides were examined utilizing a semiquantitative scoring system for GVHD evidence. Each experiment consisted of 3 to 6 mice per group.

**Flow cytometry assays**

For flow cytometry assay the cells suspension were first incubated in blocking solution CD16/32 (1:200) in FACS buffer (PBS containing 2% of FBS and 2mM of EDTA) for 30 minutes at 4ºC, and then stained with a standard panel of immunophenotyping antibodies, fluorochrome-conjugated antibodies used for flow cytometric analysis are listed in supplemental Table 1. Intracellular Foxp3 assay was performed using the Foxp3 staining kit (eBioscience) according to the manufacturer´s instruction. For intracellular cytokine staining, the cells were cultured with 50 ng/ml of 25ng/mL phorbol 12-myristate 13-acetate (PMA, Sigma-Aldrich), 1µ/mL of ionomycin (50 mg/mL), 10µg/mL of brefeldin A (Sigma) and with LPS (10 μg/ml, *Escherichia coli* serotype 0111: B4, Sigma) for 4 hours. The cells were surface labeled and processed for intracellular staining. The cytokines were assessed via cytofix/cytoperm kit. For cell viability, the cells were collected and stained with different mAbs, 7AAD, and Annexin V-PE using the PE Annexin V Apoptosis Detection Kit (BD Pharmingen, San Jose, CA) according to manufacturer`s instructions.

Data acquisition was performed on a FACSCanto flow cytometer (BD) using the FACS DIVA software program (BD Biosciences, San Jose, CA, USA) and analyzed using the Infinicyt software (Cytognos, Salamanca, Spain)

Supplementary Table 1: Fluorochrome-conjugated antibodies used for flow cytometric

| **Antibody** | **Fluorophore** | **Clone** | **Supplier** |
| --- | --- | --- | --- |
| CD62L | APC-H7 | MEL-14 | BD |
| CD19 | BV421 | 6D5 | Biolegend |
| CD11b | PE | M1/70 | Immunostep |
| CD3 | PerCP-Cy 5.5 | 145-2C11 | BD |
| CD45 | APC | 30-F11 | BD |
| Ly6G | APC-H7 | 1A8 | BD |
| CD44 | BV421 | IM7 | BD |
| CD45 | BV540 | 30-F11 | Biolegend |
| CD8 | PE | 53-6.72 | Immunostep |
| CCR7/CD197 | PE Cy7 | 4B12 | Biolegend |
| CD4 | APC | RM4-5 | BD |
| CD4 | BV421 | GK1.5 | BD |
| CD25 | APC | 3C7 | Biolegend |
| CD45 | APC-H7 | 30-F11 | BD |
| CD25 | PE | PC61.5 | Biolegend |
| CD45 | PE-Cy7 | 30-F11 | BD |
| F4/80 | BV421 | T45-2342 | BD |
| CD64 | PE | X54-5/7.1 | Biolegend |
| MHCII | PerCP-Cy 5.5 | M5/114.15.2 | BD |
| CD11b | PE Cy7 | M1/70 | BD |
| CD11c | APC | HL3 | BD |
| GR-1 | APC-H7 | RB6-8C5 | BD |
| CD206 | PE | C068C2 | Biolegend |
| CD24 | APC | M1/69 | BD |
| CD11c | APC-H7 | N418 | Biolegend |
| B220 | PE-Cy7 | RA3-6B2 | BD |
| CD5 | PerCP-Cy 5.5 | 53-7.3 | Biolegend |
| TCR-B | PE-Cy7 | H57-597 | Biolegend |
| CD21 | BV421 | 7,00E+09 | Biolegend |
| CD268 | PE | 7H22-E16 | Biolegend |
| IgM | APC | II/41 | BD |
| IgD | PerCP-Cy 5.5 | 11-26c.2a | BD |
| CD23 | APC-H7 | B3B4 | Biolegend |
| IL-10 | BV421 | JES5-16E3 | Biolegend |
| CD5 | PE | 53-7.3 | BD |
| CD1d | PerCP-Cy 5.5 | 1B1 | Biolegend |
| CD38 | APC | 90 | Biolegend |
| TCRy/B | APC | GL3 | Biolegend |
| CD8 | BV421 | 53-6.7 | BD |
| CD24 | PE | M1/69 | BD |
| CD16/32 | PURIFIED | 93 | Biolegend |
| GR-1 | FITC | RB6-8C5 | Immunostep |
| CD4 | BV510 | RM4-5 | BD |

**Supplementary Figure**


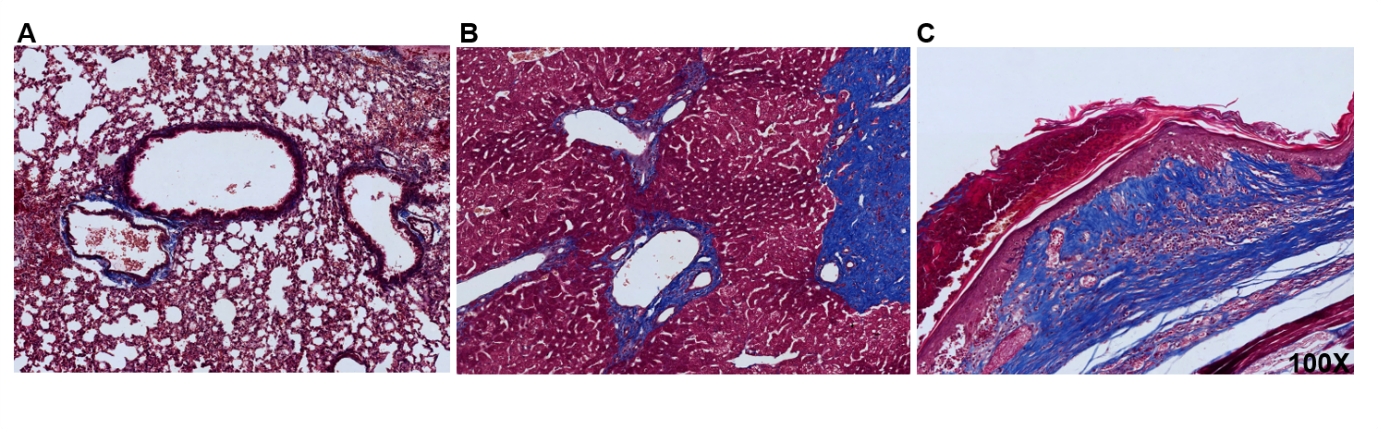


**Supplementary Figure 1:** Masson trichrome staining was used to evaluate fibrosis of the lung (A), liver (B) and skin (C) tissues, 100X.


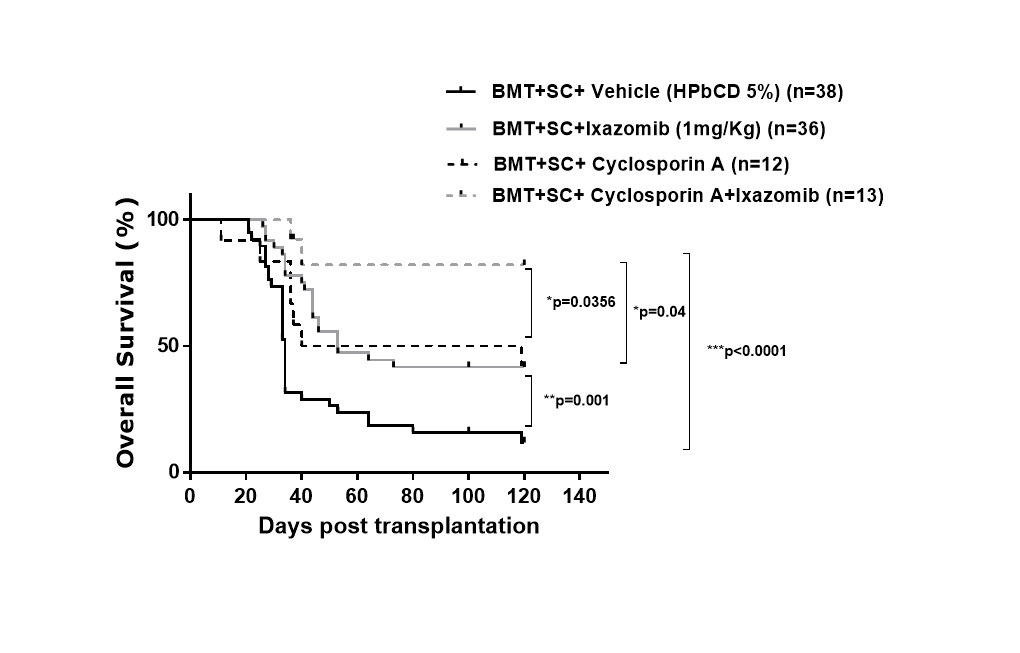


**Supplementary Figure 2:** Survival curves of mice receiving IXZ, cyclosporine A (at 5mg/Kg, intraperitoneally, 5 days a week from d -1 and maintained until 120 post transplantation) or the combination of both. Data are collected from 3 independent experiments with 4-5 mice per group. **P* < .05, ***P* < .01, and ****P* < .001 were considered significant. BMT: bone marrow transplantation; SC: splenocytes.

**
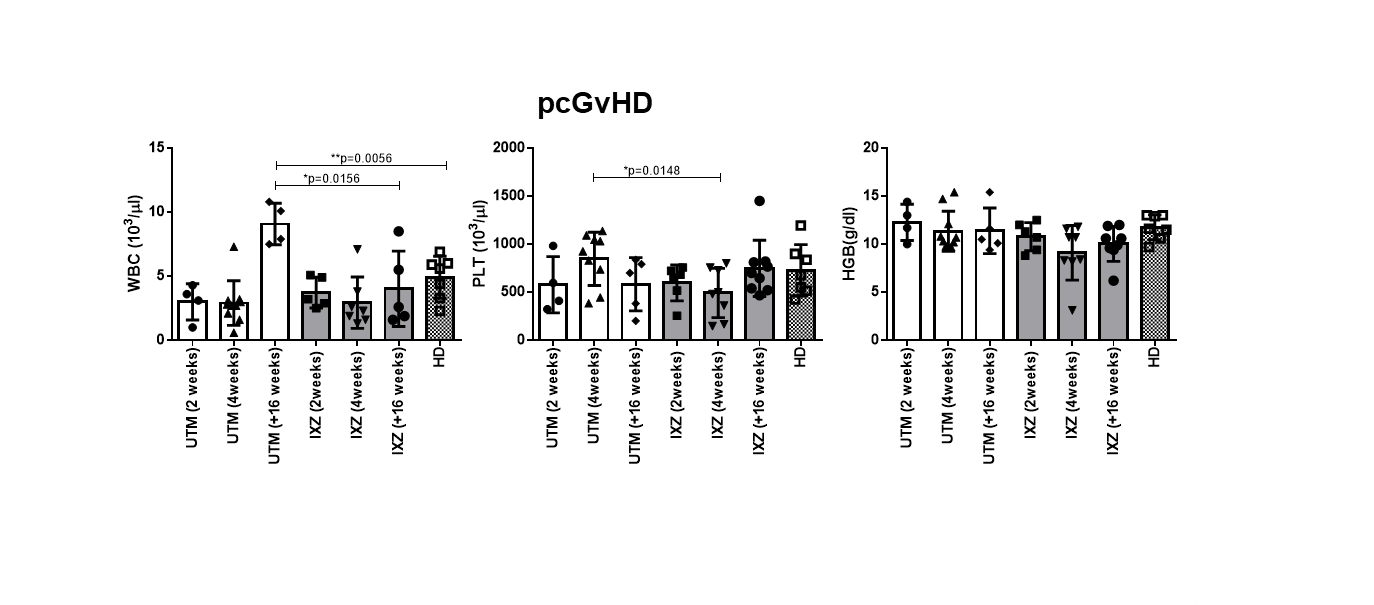
Supplementary Figure 3:** Blood cell counts of mice from pcGvHD model. N=5-10 per group. Plots represent the mean ± SEM. Statistical significance: *P* < .05, ** *P* < .01, and *** *P* < .001. UTM: untreated transplanted mice (vehicle) HD: heathy donor; SYG: syngeneic group; w: weeks after treatment.

**
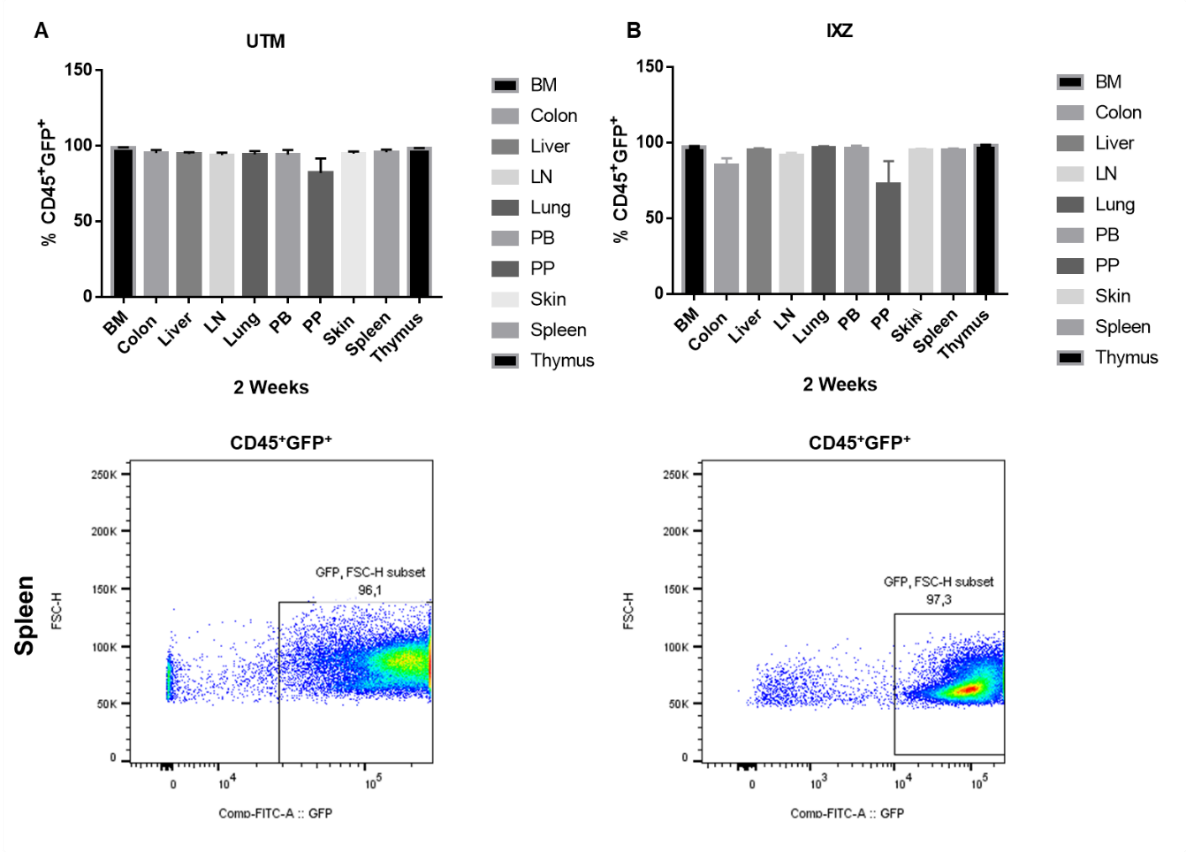
**

**Supplementary Figure 4:** Chimerism analysis of mice from pcGvHD (A) UTM (B) IXZ. Dot plot analysis of the donor cells (GFP^+^ cells) on the hematopoietic cells (CD45^+^). UTM: untreated transplanted mice (vehicle) HD: heathy donor; SYG: syngeneic group; w: weeks after treatment.


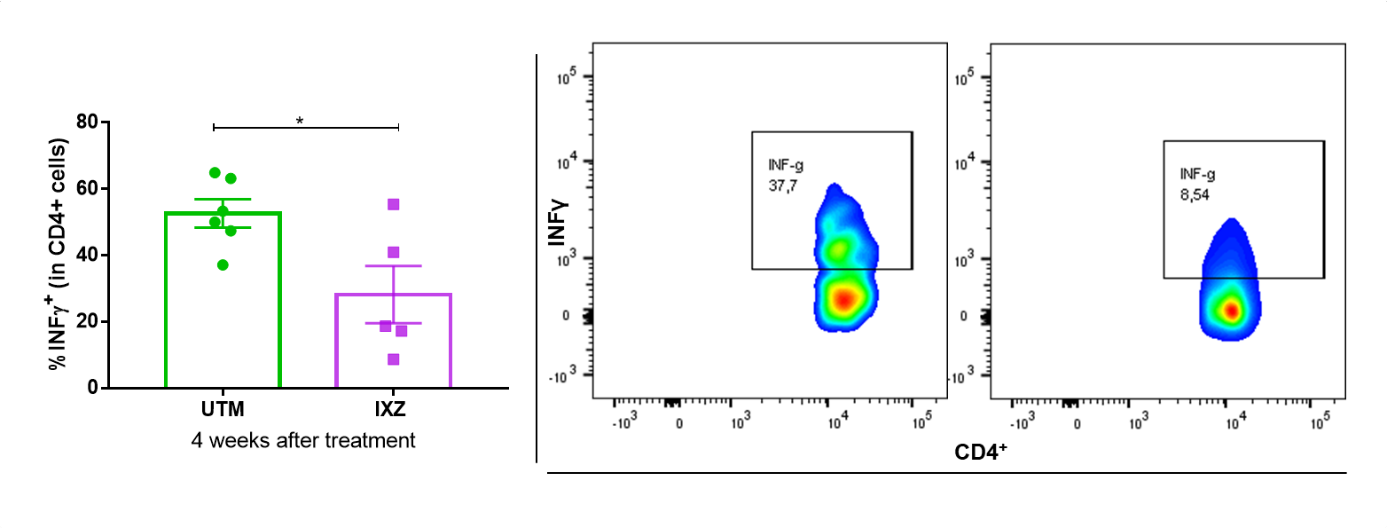


**Supplementary Figure 5:** Analysis of IFN-γ in the CD4+ T cell population in the spleen of the mice treated with Ixazomib. Data are shown as mean ± SEM. Data are collected from 4-5 independent experiments with 5-6 mice per group. * *P* < .05. UTM: untreated transplanted mice; IXZ: Ixazomib group; UTM: untreated transplanted mice (vehicle treatment).
